# Fostering Civil Discourse Across Generations: Intergenerational Dialogue on Social Justice Through Art

**DOI:** 10.1093/geroni/igaf122.3244

**Published:** 2025-12-31

**Authors:** Kozbi Bayne, Rouida Siddiqui, Thomas Lu, Ruy Tobar-Mosqueira, Todd Prusin, Madeleine Hackney

**Affiliations:** Emory School of Medicine, Atlanta, Georgia, United States; Emory School of Medicine, Atlanta, Georgia, United States; Emory School of Medicine, Atlanta, Georgia, United States; Emory Primate Center, Atlanta, Georgia, United States; Emory School of Medicine, Atlanta, Georgia, United States; Emory School of Medicine, Atlanta, Georgia, United States

## Abstract

Little is known about generational differences and similarities in beliefs about social justice. Science Gallery Atlanta (SGA) is a museum integrating science and art to foster dialogue about challenging topics, including social justice. Previous research suggests intergenerational communication about social and political issues could positively impact older and younger adults. This study investigated perceptions of justice among a diverse sample of older (65+ years) and younger (18–26 years) adults through intergenerational dialogue on justice-focused art offered through SGA’s JUSTICE exhibition. This exploratory pilot study recruited ten randomized older-younger dyads who received a mediated tour of JUSTICE. Attitudes about discrimination, equity, and activism were assessed pre- and post-tour through self-report measures, including the Belief in a Just World, Addiction Belief, and Everyday Discrimination Scales, concurrent interviews, and focus groups. Qualitative data were recorded, transcribed, and analyzed thematically using a nodal framework. Pre and post-tour, older adults were more confident than their younger counterparts about the involvement of others in social justice work. Post-tour, young adults rated community involvement in social justice work more favorably and expressed greater support for activism in daily life (p = 0.05). Interestingly, younger adults reported experiencing greater discrimination than older adults pre and post-tour (p < 0.05). Based on qualitative results, intergenerational bridging may help young adults trust in community activism’s efficacy and help older adults understand technology’s potential role in social change. Emergent themes illuminate generational differences and similarities in perceptions of social justice, suggesting SGA’s interdisciplinary experience effectively promotes discourse across different age groups.

